# Supplementary material for: Transgenerationally Transmitted DNA Demethylation of a Spontaneous Epialleles Using CRISPR/dCas9-TET1cd Targeted Epigenetic Editing in Arabidopsis
Source: Int J Mol Sci. 2022 Sep 10;23(18):10492. doi: 10.3390/ijms231810492 (PMC9504898; doi:10.3390/ijms231810492)
Supplement: Supplementary file 1 [file ijms-23-10492-s001.zip › ijms-1872536-supplementary/Supplementary File/Figure S1.pdf]

```
Bor 4      1 GTTTTTTTTTTTTTTTAACAACTACTACTACAAGTTTATTAGATTGTATAAGGTTGAT   57
Lp2-2      1 GTTTTTTTTTTTTTTTAACAACTACTACTACAAGTTTATTAGATTGTATAAGGTTGAT   57
Bs 1       1 GTTTTTTTTTTTTTTTAACAACTACTACTACAAGTTTATTAGATTGTATAAGGTTGAT   57
Kort 0     1 GTTTTTTTTTTTTTTTAACAACTACTACTACAAGTTTATTAGATTGTATAAGGTTGAT   57

Bor 4     58 ACAAATAAGAGAGTAAGAAACGAGGAAGAACAATAAAGGACTGATACAGAGTAGTAG  114
Lp2-2     58 ACAAATAAGAGAGTAAGAAACGAGGAAGAACAATAAAGGACTGATACAGAGTAGTAG  114
Bs 1      58 ACAAATAAGAGAGTAAGAAACGAGGAAGAACAATAAAGGACTGATACAGAGTAGTAG  114
Kort 0    58 ACAAATAAGAGAGTAAGAAACGAGGAAGAACAATAAAGGACTGATACAGAGTAGTAG  114

Bor 4    115 TAGACAAAACATATTGAGCCATCTTATCTGCAGCACTATTTCGTTCACGATTGATGT  171
Lp2-2    115 TAGACAAAACATATTGAGCCATCTTATCTGCAGCACTATTTCGTTCACGATTGATGT  171
Bs 1     115 TAGACAAAACATATTGAGCCATCTTATCTGCAGCACTATTTCGTTCACGATTGATGT  171
Kort 0   115 TAGACAAAACATATTGAGCCATCTTATCTGCAGCACTATTTCGTTCACGATTGATGT  171

Bor 4    172 GGAGAAGAGTGACGTTGGATAGTTTTGCCATCCAATGACGAATATCGAACAGGAGAG  228
Lp2-2    172 GGAGAAGAGTGACGTTGGATAGTTTTGCCATCCAATGACGAATATCGAACAGGAGAG  228
Bs 1     172 GGAGAAGAGTGACGTTGGATAGTTTTGCCATCCAATGACGAATATCGAACAGGAGAG  228
Kort 0   172 GGAGAAGAGTGACGTTGGATAGTTTTGCCATCCAATGACGAATATCGAACAGGAGAG  228

Bor 4   1369 CTAAACGAACTCGTAATCCTTGACAAAGTAACTCTTTTCCATGTTGTATAGAACGCC 1425
Lp2-2   1369 CTAAACGAACTCGTAATCCTTGACAAAGTAACTCTTTTCCATGTTGTATAGAACGCC 1425
Bs 1    1369 CTAAACGAACTCGTAATCCTTGACAAAGTAACTCTTTTCCATGTTGTATAGAACGCC 1425
Kort 0  1369 CTAAACGAACTCGTAATCCTTGACAAAGTAACTCTTTTCCATGTTGTATAGAACGCC 1425

Bor 4   1426 ATCCAAAAGATGTATAGGTTCCTGTCACCGCTTCCAAATAAGAGGTTTGAGGATGAT 1482
Lp2-2   1426 ATCCAAAAGATGTATAGGTTCCTGTCACCGCTTCCAAATAAGAGGTTTGAGGATGAT 1482
Bs 1    1426 ATCCAAAAGATGTATAGGTTCCTGTCACCGCTTCCAAATAAGAGGTTTGAGGATGAT 1482
Kort 0  1426 ATCCAAAAGATGTATAGGTTCCTGTCACCGCTTCCAAATAAGAGGTTTGAGGATGAT 1482

Bor 4   1483 ACATACCTTTATAAAGACGGGCCAAAAGACTCTGAGGATTTCTCAAAATTCTCCAAG 1539
Lp2-2   1483 ACATACCTTTATAAAGACGGGCCAAAAGACTCTGAGGATTTCTCAAAATTCTCCAAG 1539
Bs 1    1483 ACATACCTTTATAAAGACGGGCCAAAAGACTCTGAGGATTTCTCAAAATTCTCCAAG 1539
Kort 0  1483 ACATACCTTTATAAAGACGGGCCAAAAGACTCTGAGGATTTCTCAAAATTCTCCAAG 1539

Bor 4   2395 CTACCCGATCATAGGCCTTGCTTATATCAGTTTTAATCGCCAAATACCCATTCTGGC 2451
Lp2-2   2395 CTACCCGATCATAGGCCTTGCTTATATCAGTTTTAATCGCCAAATACCCATTCTGGC 2451
Bs 1    2395 CTACCCGATCATAGGCCTTGCTTATATCAGTTTTAATCGCCAAATACCCATTCTGGC 2451
Kort 0  2395 CTACCCGATCATAGGCCTTGCTTATATCAGTTTTAATCGCCAAATACCCATTCTGGC 2451

Bor 4   2452 ACTCCCTCTTCGACTTGAGTGAATGTAGAAGTTCATGAGCCACTAGTACATTGTCAG 2508
Lp2-2   2452 ACTCCCTCTTCGACTTGAGTGAATGTAGAAGTTCATGAGCCACTAGTACATTGTCAG 2508
Bs 1    2452 ACTCCCTCTTCGACTTGAGTGAATGTAGAAGTTCATGAGCCACTAGTACATTGTCAG 2508
Kort 0  2452 ACTCCCTCTTCGACTTGAGTGAATGTAGAAGTTCATGAGCCACTAGTACATTGTCAG 2508

Bor 4   2509 ATATATTACGACCAGGGACAAAGGCTGCCTGAGAATCTGAGATTATAACTCCCAAAC 2565
Lp2-2   2509 ATATATTACGACCAGGGACAAAGGCTGCCTGAGAATCTGAGATTATAACTCCCAAAC 2565
Bs 1    2509 ATATATTACGACCAGGGACAAAGACTGCCTGAGAATCTGAGATTATAACTCCCAAAC 2565
Kort 0  2509 ATATATTACGACCAGGGACAAAGGCTGCCTGAGAATCTGAGATTATAACTCCCAAAC 2565

Bor 4   2566 AATCTGAGA                                                 2574
Lp2-2   2566 AATCTGAGA                                                 2574
Bs 1    2566 AATCTGAGA                                                 2574
Kort 0  2566 AATCTGAGA                                                 2574
```

Figure S1 Result of NMR19-4 sequence alignment and information of sgRNA sequence, position and PAM sequence in Bor 4, Lp2-2, Bs 1 and Krot 0. Yellow represents sgRNA and pink represents PAM sequence.
