# Supplementary material for: Transgenerationally Transmitted DNA Demethylation of a Spontaneous Epialleles Using CRISPR/dCas9-TET1cd Targeted Epigenetic Editing in Arabidopsis
Source: Int J Mol Sci. 2022 Sep 10;23(18):10492. doi: 10.3390/ijms231810492 (PMC9504898; doi:10.3390/ijms231810492)
Supplement: Supplementary file 1 [file ijms-23-10492-s001.zip › ijms-1872536-supplementary/Supplementary File/Figure S2.pdf]

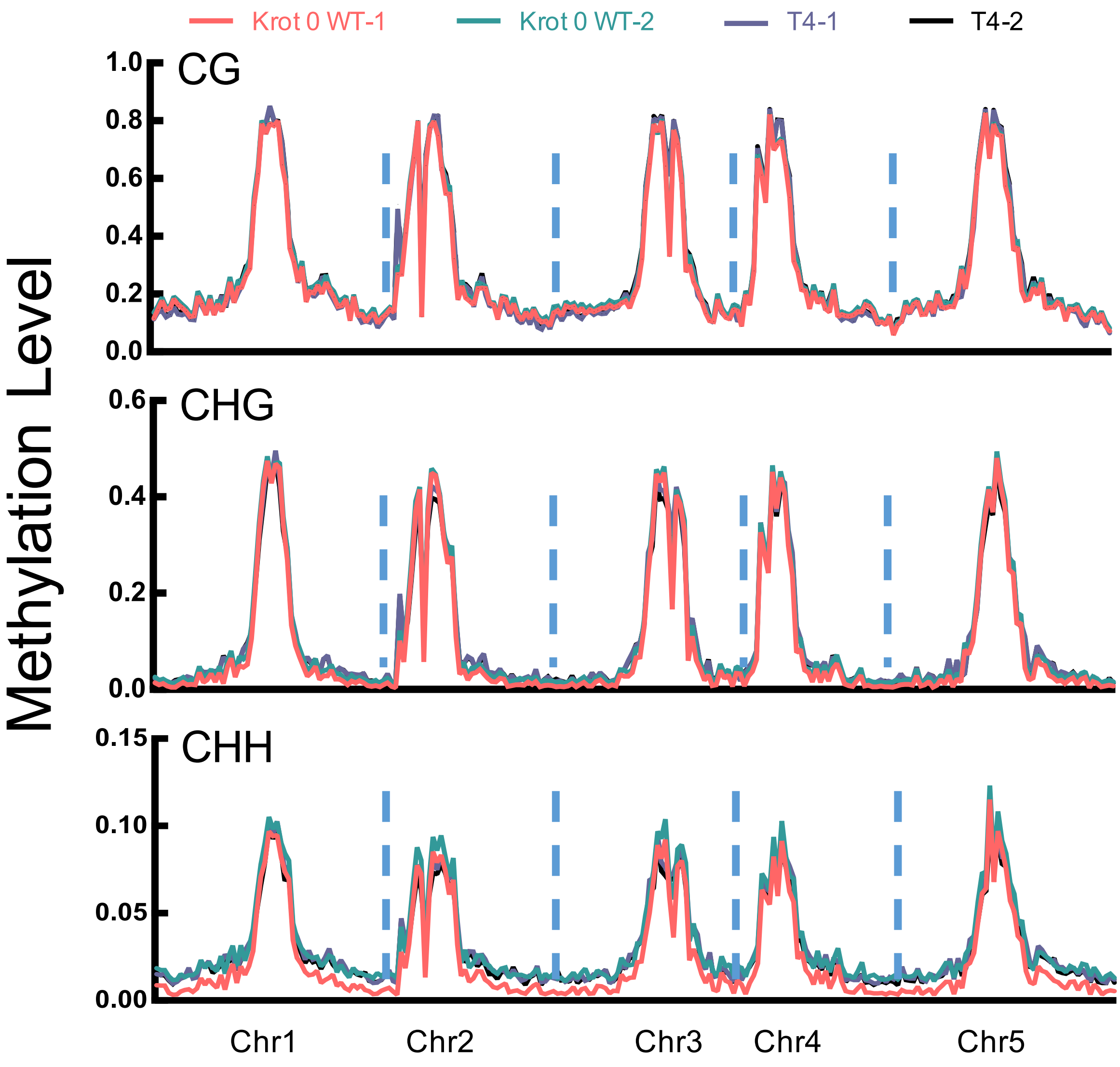

Figure S2 NMR19-4 related CRISPR/dCas9-TET1cd does not affect the genome methylation levels. Genome methylation of CG, CHG and CHH in two indepent Krot 0 WT and T4.
