# Supplementary material for: Transgenerationally Transmitted DNA Demethylation of a Spontaneous Epialleles Using CRISPR/dCas9-TET1cd Targeted Epigenetic Editing in Arabidopsis
Source: Int J Mol Sci. 2022 Sep 10;23(18):10492. doi: 10.3390/ijms231810492 (PMC9504898; doi:10.3390/ijms231810492)
Supplement: Supplementary file 1 [file ijms-23-10492-s001.zip › ijms-1872536-supplementary/Supplementary File/Figure S3.pdf]

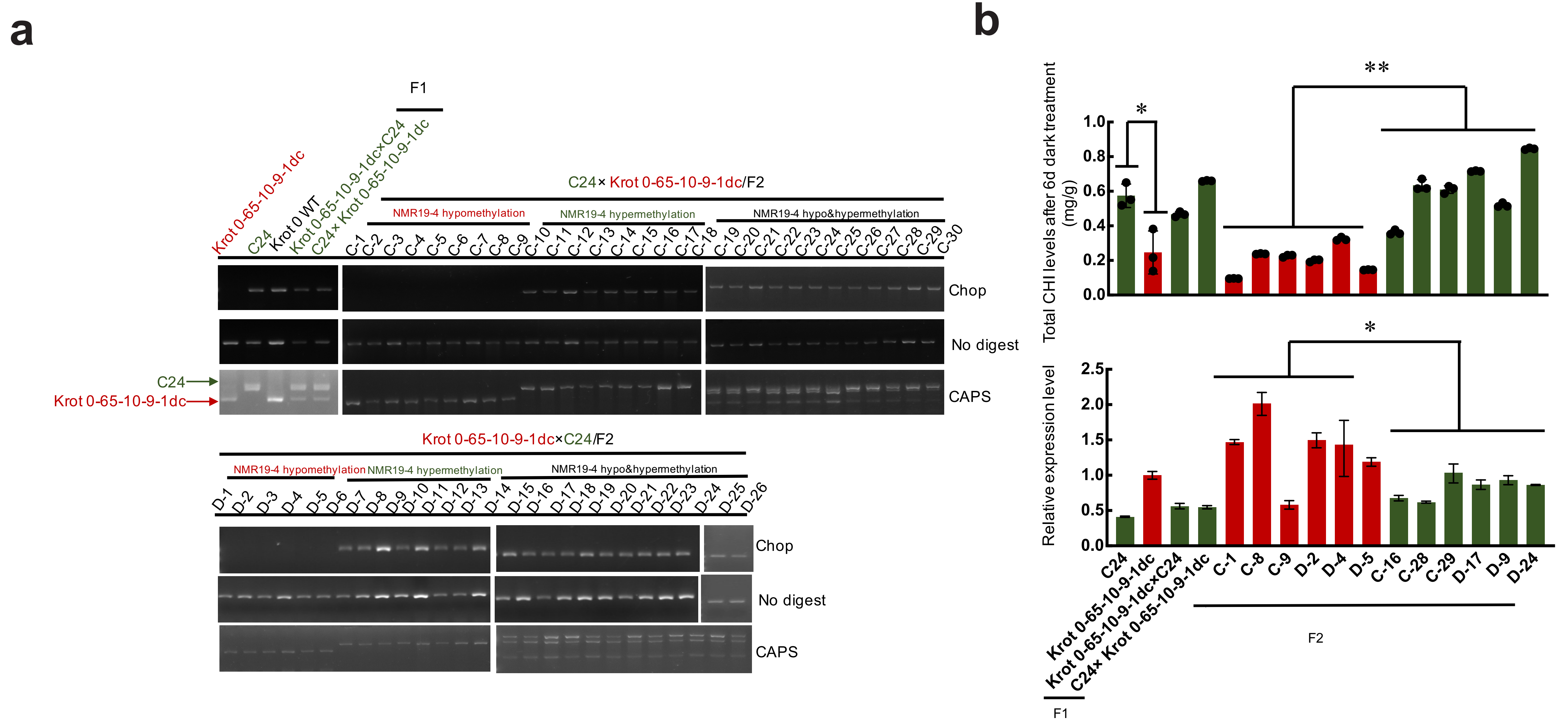

Figure S3 The trans-generational transmitted by Mendelian inheritance in F1 hybrid and F2 segregation. a The methylation status of NMR19-4 was detected by Chop-PCR in F1 and F2 recombinant lines. The arrows at the left side of the gel photo indicate the allele corresponding to the band in the gel. The numbers of C1 to C30 and D1 to D26 represent the different individual plants and all of them were used for Chop-PCR, CAPS. **b** Quantification of chlorophyll content after 6 days dark induction in F1 and F2 recombinant lines (n=3) (*,p<0.05, t-test)(**,p<0.01,t-test).The expression level of *PPH* in F1 and F2 recombinant lines by qPCR.
