# Supplementary material for: Transgenerationally Transmitted DNA Demethylation of a Spontaneous Epialleles Using CRISPR/dCas9-TET1cd Targeted Epigenetic Editing in Arabidopsis
Source: Int J Mol Sci. 2022 Sep 10;23(18):10492. doi: 10.3390/ijms231810492 (PMC9504898; doi:10.3390/ijms231810492)
Supplement: Supplementary file 1 [file ijms-23-10492-s001.zip › ijms-1872536-supplementary/Supplementary File/Figure S4.pdf]

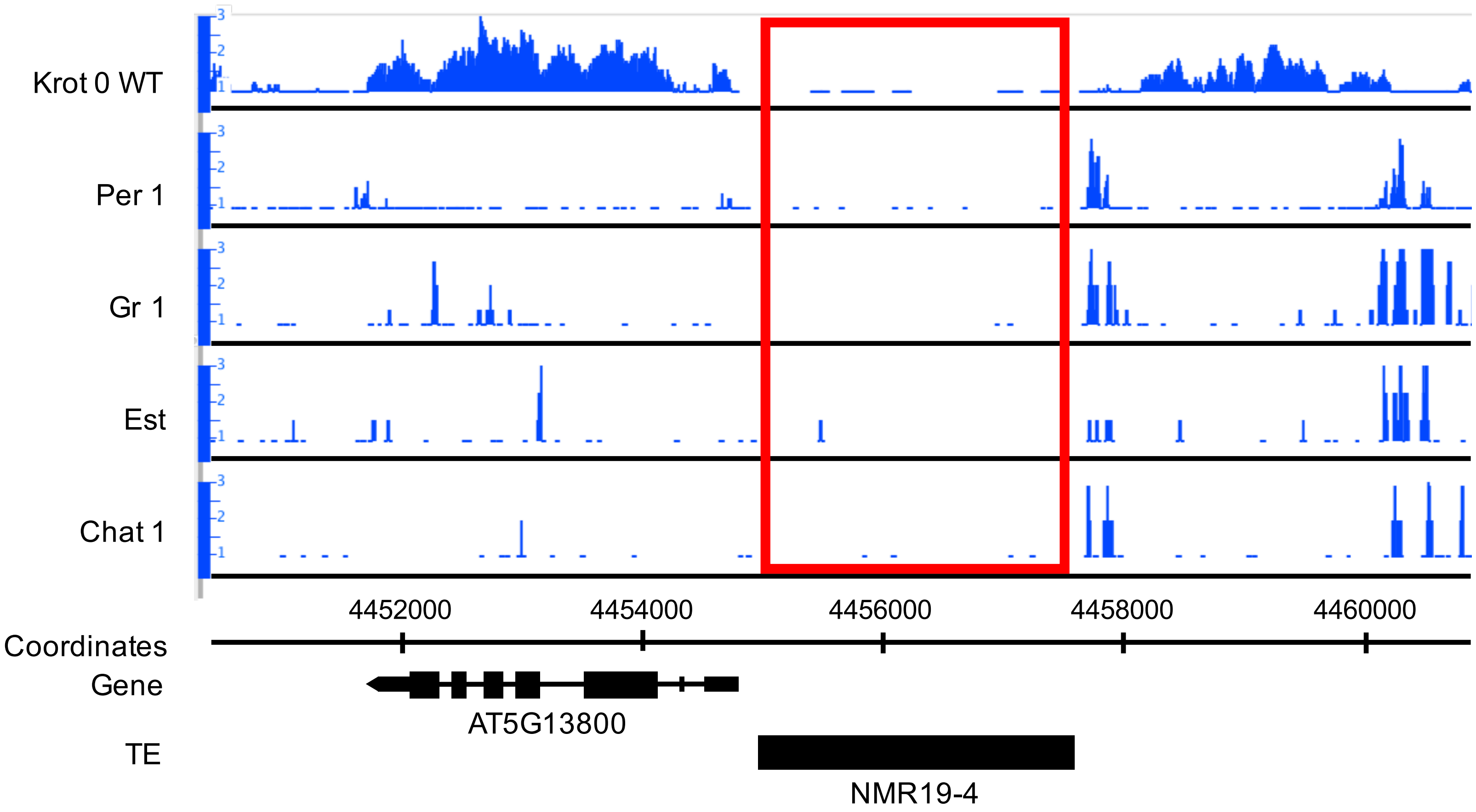

Figure S4 ATAC-seq of Krot 0 WT, Per 1, Chat 1,Gr 1 and Est. NMR19-4 of Krot 0 WT and Per 1 were hypermethylation. NMR19-4 of Chat 1,Gr 1 and Est were hypomethylation. Red rectangle represents NMR19-4.
